# Is Drp1 a link between mitochondrial dysfunction and inflammation in Alzheimer’s disease?

**DOI:** 10.3389/fnmol.2023.1166879

**Published:** 2023-05-12

**Authors:** Oualid Sbai, Veronica Bazzani, Shreya Tapaswi, Joshua McHale, Carlo Vascotto, Lorena Perrone

**Affiliations:** ^1^Institut Pasteur de Tunis, LR11IPT02, Laboratory of Transmission, Control and Immunobiology of Infections (LTCII), Tunis, Tunisia; ^2^Department of Medicine, University of Udine, Udine, Italy; ^3^IMol Polish Academy of Sciences, Warsaw, Poland; ^4^Department of Advanced Medical and Surgical Sciences, University of Campania Luigi Vanvitelli, Naples, Italy

**Keywords:** Alzheimer inflammation, mitochondria, DRP1, NLRP3, TXNIP

## Abstract

Recent advances highlight that inflammation is critical to Alzheimer Disease (AD) pathogenesis. Indeed, several diseases characterized by inflammation are considered risk factors for AD, such as type 2 diabetes, obesity, hypertension, and traumatic brain injury. Moreover, allelic variations in genes involved in the inflammatory cascade are risk factors for AD. AD is also characterized by mitochondrial dysfunction, which affects the energy homeostasis of the brain. The role of mitochondrial dysfunction has been characterized mostly in neuronal cells. However, recent data are demonstrating that mitochondrial dysfunction occurs also in inflammatory cells, promoting inflammation and the secretion of pro-inflammatory cytokines, which in turn induce neurodegeneration. In this review, we summarize the recent finding supporting the hypothesis of the inflammatory-amyloid cascade in AD. Moreover, we describe the recent data that demonstrate the link between altered mitochondrial dysfunction and the inflammatory cascade. We focus in summarizing the role of Drp1, which is involved in mitochondrial fission, showing that altered Drp1 activation affects the mitochondrial homeostasis and leads to the activation of the NLRP3 inflammasome, promoting the inflammatory cascade, which in turn aggravates Amyloid beta (Ab) deposition and tau-induced neurodegeneration, showing the relevance of this pro-inflammatory pathway as an early event in AD.

## 1. Introduction

Alzheimer’s disease (AD) is the most common type of dementia, with prevalence rates of 11% in those of 65 years and older (Hebert et al., 2013) and 68% in memory disorder clinics (Paulino Ramirez Diaz et al., 2005). With the progression of the disease, macroscopic atrophy affects the entorhinal area and hippocampus, amygdala, and associative regions of the neocortex. AD is characterized by white matter loss and myelin degeneration due to death of oligodendrocytes occurring in the early phase of AD. Hallmark signs of AD are the formation of amyloid plaques and neurofibrillary tangles (NFT) in the hippocampal and entorhinal regions (Perrone et al., 2012). Amyloid plaques are constituted by accumulation of the beta amyloid peptides (Aβ), which aggregate both intracellularly and extracellularly and is produced by the processing of the amyloid precursor protein (APP; Hardy and Selkoe, 2002). NFT are formed in neurons by intracellular aggregation of hyperphosphorylated tau. About 90–95% of AD cases are sporadic and only 5–10% are familiar, showing mutations in APP gene or the genes encoding the proteins involved in APP cleavage and Aβ production (presenilin 1-PSEN1-and presenilin 2 PSEN2). The presence of mutations in APP or PSEN1/2 in familiar AD initially supported the hypothesis of the “Amyloid cascade” as a central player in AD onset and progression, indicating the over-production of Aβ as the causative event responsible for the pathophysiological process leading to AD progression. However, recent studies underline that Aβ deposition and NFT are not sufficient to clarify AD onset and progression, opening the way to the amyloid-inflammatory cascade (Wang and Mandelkow, 2016; Long and Holtzman, 2019). In agreement, aging and several diseases that are risks for AD, such as type 2 diabetes, obesity, hypertension, and metabolic syndrome, are characterized by chronic inflammation (Matrone et al., 2015). It has been hypothesized a central role of microglia activation as initial step initiating the pathological cascade leading to AD. Indeed, several AD risk genes play a central role in the innate immunity (Scheltens et al., 2021). Microglia are central for the brain homeostasis, ensuring effective synapse pruning and plasticity, as well as supporting myelin stability. Several microglia phenotypes have been described, showing different morphology, molecular and metabolic characteristics that correspond to the different functions of the microglia. Recent data underline that dysfunctional microglia affect the synaptic plasticity and alter the cognitive function, contributing to AD pathophysiology. Interestingly, mitochondrial fission promotes inflammatory activation, showing a link between mitochondrial dynamics and microglia activation (Lawrence et al., 2022). NOD-like receptor family, pyrin domain containing 3 (NLRP3) plays a central role in inflammation, by producing pro-inflammatory cytokines. On the other hands, Dynamin Related Protein 1 (Drp1) is essential for the homeostasis of mitochondria dynamics and is implicated in mitochondria fission. We will summarize below the link between Drp1 and NLRP3 in promoting inflammation and participating in AD pathophysiology.

## 2. AD and inflammation

In the last decades, increasing evidence has demonstrated that a sustained immune response can be classified as an essential factor involved in AD pathophysiology as well as Aβ aggregation and tau hyperphosphorylation (Matrone et al., 2015; Kinney et al., 2018). In normal conditions, acute inflammation is a response counteracting injury to the brain. This well-established mechanism serves as protection and may be activated upon different stimuli. However, the disruption of the equilibrium between pro-inflammatory and anti-inflammatory signaling can results in chronic neuroinflammation, where the sustained immune response becomes a central feature of neurodegenerative disorders (Matrone et al., 2015). Recent studies underline that inflammation plays a central role in AD pathophysiology and is implicated in the development of the pathological changes (Aβ aggregation and NFT) observed in AD. In agreement, various inflammatory molecules have been proposed as AD biomarkers (Novoa et al., 2022). In addition, diseases characterized by systemic inflammation, such as obesity, type 2 diabetes, and cerebrovascular diseases, are considered risk factors for AD (Novoa et al., 2022). In neurodegenerative diseases, inflammation can be produced in two ways. In one mechanism, peripheral inflammation produces cytokines, which alters and cross the Blood Brain Barrier (BBB), further inducing the release of pro-inflammatory factors by the brain endothelial cells and by glial cells associated to the BBB. This process enhances the BBB permeability to peripheral immune cells, leading to the entry of leucocytes into the brain (Varatharaj and Galea, 2017). These events promote the activation of astrocytes and microglia, leading to further production of cytokines into the brain, ultimately promoting neuronal dysfunction. This mechanism is defined as neuroinflammation (Carson et al., 2006). In a second mechanism, the innate immune system of the brain (such as the microglia) promotes a cascade leading to neuroinflammation. This mechanism can be induced by neuronal lesions or aggregated proteins, such as Aβ, which activates the astro-glial cells, leading to cytokine production and ultimately promoting synaptic dysfunction and neurodegeneration (Lull and Block, 2010).

As with most of the other mechanisms investigated in AD, it is not yet definitively understood whether inflammation is the cause, contribution, or secondary phenomenon of this disorder. In the next paragraphs, we will delineate a comprehensive report of the studies performed to elucidate the role of inflammation and the new frontiers aimed to target inflammation in AD.

### 2.1. Role of inflammation in AD: clinical data

The majority age related diseases, such as diabetes, and obesity-that are risk factors for AD-and AD are characterized by chronic inflammation (Chung et al., 2009). In the 1980s, it has been demonstrated the presence of inflammatory proteins and immune-related cells in the proximity of Aβ plaques (Rogers et al., 1988; Griffin et al., 1989). Since the 1990s, researchers have demonstrated a significant presence of sustained inflammation in patients with AD (Aisen and Davis, 1994), confirmed by post-mortem tissues analysis (Gomez-Nicola and Boche, 2015). Epidemiological studies of large-scale cohorts have shown that people showing enhanced pro-inflammatory proteins in the blood in mid-life are at higher risk of cognitive decline over the decades compared to subjects maintaining a low presence of pro-inflammatory factors in the blood (Leung et al., 2013; Huang et al., 2022). In addition, in their later life, these individuals are characterized by lower volume of brain with abnormal microstructure of white matter, increased myelin loss and the inability of the oligodendrocytes, the cells responsible for the production and maintenance of myelin, to repair myelin damages (Nasrabady et al., 2018; Collij et al., 2021). These observations were among the first to support the idea that systemic inflammation occurs one or two decades before the appearance of dementia symptoms, suggesting its active role in promoting the progression of cognitive decline and neurodegeneration. In parallel, genome-wide association studies (GWAS) reported that more than 60% of the genes linked to late-onset sporadic AD are inflammation-related (Efthymiou and Goate, 2017; Misra et al., 2018; Carpanini et al., 2021). In this scenario, preliminary studies aimed to evaluate the potential beneficial effect of long-term administration of anti-inflammatory drugs. Indeed, people who regularly took anti-inflammatory drugs over a long period of time showed reduced risk of developing AD later in life. Unfortunately, the search for anti-inflammatory drugs effective in preventing AD was less straight forward than it could seem looking at the plethora of data supporting the pivotal role of inflammation. Indeed, the effect of anti-inflammatory drugs remains under debate. Chronic inflammation exacerbates amyloid β deposition and tau hyperphosphorylation and participate to the pathogenesis of AD (Matrone et al., 2015). Interestingly, chronic administration of non-steroidal anti-inflammatory drugs (NSAIDs) appears to be beneficial only in the very early stages of the AD process along with initial Aβ deposition, early microglia activation and subsequent release of pro-inflammatory mediators (Imbimbo et al., 2010). On the other side, over time the beneficial effect of NSAIDs is no more significant, especially when older groups of patients are studied. In 2009, Breitner and colleagues suggested that the differences seen in these observational studies may be due to the fact that NSAIDs may not simply reduce the risk of AD, but delay AD onset in later ages (Breitner et al., 2009), suggesting that NSAIDs may be beneficial in delaying AD only when administered in the young age. Since AD is more common in old subjects and occurs as a chronic effect after decades from the initial pathological alteration, NSAID is beneficial in preventing AD when administered to young subject, while NSAID is less effective when administered in the old age, explaining the variance observed among different patient groups. Other factors may also cause the inconsistencies reported in several publications. The selection of the most effective NSAIDs for a study and the presence of other factors as concomitant pathologies to AD can affect the epidemiological results, producing discrepancies among different studies. To overcome these limits, in recent years, more homogeneous approaches have been taken in clinical trials to clarify the effects of NSAIDs without confounding variables. Jack Rivers-Auty and colleagues used logistic regression and an innovative approach of negative binomial generalized linear mixed modeling to investigate both prevalence and cognitive decline in the AD Neuroimaging dataset for commonly used NSAIDs and paracetamol (Rivers-Auty et al., 2020). They demonstrated that most NSAIDs can reduce the prevalence of AD, but not cognitive decline. Interestingly, paracetamol also had a similar effect, which lead the authors to hypothesize that the prevalence of AD is independent of inflammation. Finally, they also analyzed the use of diclofenac (a non-steroidal anti-inflammatory drug), finding a significant association between diclofenac intake and the reduction in AD incidence and similarly to slower cognitive decline, suggesting a possible therapeutic effect of this compound in AD. Recent genetic data strongly support the key role of inflammation and immune-related genes in AD pathogenesis by demonstrating that a mutation in the Triggering Receptor Expressed on Myeloid Cells 2 (TREM2) confers a very high likelihood to develop AD (Basha et al., 2023). The R47H allelic variant of TREM2 confers a 2–4,5-fold increased risk of developing AD (Basha et al., 2023). TREM2 variants are the second genetic risk factor for AD, behind apolipoprotein E4 (ApoE4), demonstrating the key role of the innate immunity in AD pathogenesis (Basha et al., 2023). As we have seen, clinical studies present limits that are difficult to overcome: they require large cohort of individuals, long periods of observations, they can be affected by the presence of co-morbidities, environmental factors, and habits that can alter the outcome of a study. Moreover, it is almost impossible to standardize the protocols adopted among different studies in order to compare their results. Finally, the investigations of the molecular mechanisms of inflammation involved in the pathogenesis of AD is pivotal to develop drugs blocking the chronic mechanism that support AD development. For these reasons, pre-clinical studies are crucial and mouse transgenic models (expressing mutant amyloid precursor protein and presenilin mutants, resulting in increased Aβ deposition, or human tau mutant leading to tau hyperphosphorylation) have helped the identification of the focal pathways dysregulated in neuroinflammatory diseases, as described in the next paragraph.

### 2.2. Role of inflammation in AD: pre-clinical data

Initially the observed presence of inflammation in AD patients was considered the consequence of neuronal damage, which in turn activate the immune system promoting the inflammatory response. However, recent studies demonstrate that chronic inflammation in AD enhances both Aβ- and NFT-induced pathology. Notably, recent studies underline that the amyloid cascade hypothesis is not sufficient to explain the development of NFT and suggest that inflammation may represent the link between the initial Aβ-induced dysfunction and the subsequent development of NFT. In agreement, recent investigations demonstrate that inflammation exacerbates both Aβ- and NFT-induced pathology leading to AD (Kinney et al., 2018).

Studies carried out in different animal models are essential to clarify the role of inflammation in AD. The role of pro-inflammatory factors in promoting neurodegeneration has been summarized by Chen and colleagues (Chen et al., 2016). Some researchers investigated the effect of anti-inflammatory compounds in AD. NSAIDs treatment in AD mice models ameliorates AD pathophysiology (McGeer and McGeer, 2007). Studies have also been carried out in not transgenic models of AD, further supporting the role of ani-inflammatory compounds in ameliorating AD. Indeed, Lindsay and colleagues demonstrate that andrographolide—a natural compound-ameliorates not only inflammation but also oxidative stress in *Octodon degus,* which is a rodent that develops AD spontaneously (Lindsay et al., 2020). Moreover, the effect of an anti-inflammatory compound (GsRb1) has been analyzed in a rat model of AD, where Aβ was injected intraventricularly. GsRb1 treatment ameliorates the inflammatory reaction and restored the learning capability in this AD model, further supporting the role of inflammation in AD pathology (Novoa et al., 2022). To further elucidate the pathological role of neuroinflammation in AD, different strategies using immune challenge-based models and neurotoxin-induced AD models have also been employed (Nazem et al., 2015). According to the endotoxin hypothesis, the endotoxin molecules cause or contribute to the neurodegenerative process (Brown, 2019). LPS-induced rodent models are used for studying neuroinflammation and inflammation-induced amyloidosis. These rodents develop memory impairment by affecting the consolidation of memory process. Acute treatment with LPS before training blocks contextual-cue fear conditioning, which is a hippocampal-dependent learning paradigm (Zakaria et al., 2017). These data further support the role of inflammation in promoting neuronal dysfunction (Brown, 2019). Since LPS treatment in rodents induces both amyloidosis and cognitive dysfunction, it has been proposed as AD model (Zakaria et al., 2017). However, when an LPS-induced memory impairment AD model is designed, the following factors must be taken into consideration: route of administration (mainly intraperitoneal and intracerebroventricular), duration of exposure, age, and sex of the animal. LPS administration causes many behavioral effects, namely, fever, hypersomnia, activation of hypothalamus-pituitary–adrenal (HPA) axis-causing sympathetic activation, reduction in exploration, social interaction, consumption, and activity. According to studies based on LPS injection, researchers have showed that peripheral inflammation induces neuroendocrine alterations, astrocyte and microglia activation, as well as cyclooxygenase-2 (COX-2), inducible nitric oxide synthase (iNOS) and pro-inflammatory cytokine expression in the brain (Brown, 2019). Interestingly, gut microbiome derived LPS accumulates in AD affected brain, further aggravating the pro-inflammatory environment of the brain (Zhao et al., 2021). In addition, intracellular accumulation of APP, Aβ peptide, and hyperphosphorylated tau as well as exacerbation of memory deficits were observed in LPS-treated APP transgenic mice (Sheng et al., 2003; Lee et al., 2008). However, this approach has limitations, as seen when evaluating the number of injections and the route of administrations used (directly into the central nervous system vs. systemic), which can lead to different pathological effects and contradictory results (Valero et al., 2014). The data obtained by injecting LPS in wild type mice and rats are important to demonstrate the role of inflammation in promoting neuronal dysfunction and an AD-like phenotype. More detailed analysis of the molecular pathways involved have been carried out in transgenic AD mice models, unveiling specific inflammatory pathways that are central in AD pathophysiology.

The *App ^NL-G-F^* knock in (KI) model carries a combination of Swedish, Arctic, and Iberian APP mutations. This KI model more closely represents human amyloidogenic pathways than other APP models. Another KI model used to unveil the early impact of neuroinflammation in AD is the *App ^NL-F^*. Both KI models show an Aβ plaque composition comparable to that of AD patients. Interestingly, in these model microglia is associated with diffuse plaques and mushroom spine loss, underlying the crucial function of microglia-mediated synapse loss and supporting that the expression of neuroinflammation-related genes is an AD risk factor (Saito et al., 2014).

The role of TREM2 in AD pathophysiology is well characterized in the APP/PS1 and 5xFAD AD mice models. Both heterozygous and homozygous Knock out (KO) of TREM2 in these AD models strongly reduce the presence of macrophages associated to Aβ plaques, reducing Aβ plaques load in 4 months old AD mice, while Aβ load is increased in 8 months old AD mice lacking TREM2 (Basha et al., 2023). TREM2 KO in AD mice leads also to reduced production of pro-inflammatory cytokines and ameliorates astrocytosis, as observed by decreased expression of glial fibrillary acidic protein (GFAP; Basha et al., 2023). However, microglia and macrophages lacking TREM2 show a decreased capability to phagocytose Aβ and apoptotic cells and TREM KO in AD mice finally lead to enhanced neurodegeneration (Kinney et al., 2018). TREM2 is also involved in tau-mediated pathology. Indeed, the KO of TREM2 in mice expressing human tau aggravates tau pathology (Kinney et al., 2018). Studies in AD and tau mice models suggest that TREM2 plays a dual role in Aβ and tau pathology, appearing to being beneficial in the initial phase of the disease by contributing in altered protein phagocytosis, whereas it exerts a pathological role in the later phase of the disease by enhancing inflammation and neurodegeneration (Kinney et al., 2018). This dual role of TREM2 is currently explained by three distinct mechanisms related to inflammation that are relevant in AD: (i) the function of phagocytosis of damaged and misfolded proteins; (ii) the survival and proliferation of cells involved in the inflammatory response; (iii) the regulation of the whole inflammatory process (Kinney et al., 2018).

Further supporting the role of inflammation in AD, several studies carried out in AD mice models reveal the relevance of the Receptor for Advanced Glycation Endproducts (RAGE) in AD pathophysiology (Perrone et al., 2012). RAGE is a multi-ligand receptor whose activity is also triggered by Aβ. Silencing of RAGE specifically in microglia ameliorates neuronal dysfunction in an AD contest (Origlia et al., 2010). Interestingly, High Mobility Group Box 1 (HMGB1)—another ligand of RAGE-seems to be involved in AD progression (Mo et al., 2023). HMGB1 is a ubiquitous non-histone DNA binding protein, which may exert various functions depending on its subcellular localization. Inside the nucleus, HMGB1 acts as a structural chromatin protein, regulating DNA repair and gene expression (Mo et al., 2023). In the cytoplasm, HMGB1 modulates autophagy and is implicated in the removal of damaged mitochondria (Mo et al., 2023). HMGB1 can also be secreted by inflammatory cells or released by necrotic cells. Extracellular HMGB1 is considered as an alarm protein or a damage associated molecular pattern (DAMP) protein and induces inflammation through interaction with various receptors, such as RAGE, Toll-like receptor 4 (TLR 4), CD24, and CXCR4 (Mo et al., 2023). Inhibition of HMGB1 is beneficial in AD mice (Paudel et al., 2020), further supporting the key role of inflammation in AD onset and progression.

## 3. Cell types promoting inflammation in AD

### 3.1. Microglia

Microglia are known resident immune cells within the central nervous system (CNS) and are among the main responsible for the surveillance of the surrounding neurons health. Among the various microglia phenotypes described, three of them are more relevant for the understanding of AD pathophysiology: the steady state, the activated and the primed phenotype (Bivona et al., 2023). Steady state microglia exert a neuroprotective function and present a basic and low level of cytokine production, with an anti-inflammatory function. Activated microglia show a protective function against injuries, enhances the production of cytokines, both pro-inflammatory (IL-1β, IL-18, and TNF) and anti-inflammatory cytokines in order to counteract an excessive inflammation and inhibit a subsequent neuronal damage. Activated microglia can revert its phenotype to steady state, when the activating stimulation is removed. Primed microglia show a more aggressive and pro-inflammatory activity and maintain the memory of the specific stimulation with a Toll-like receptor 4 (TLR4)-mediated mechanism. Primed microglia cannot revert his phenotype and turn again on the steady state phenotype (Bivona et al., 2023).

Microglial activation is highly regulated. In normal conditions, the microglia are maintained inactive by healthy neurons through the continuous release of inhibitors such as the chemokine CX3CL1, whose receptor CX3CR1 is expressed uniquely on microglia surface (Arnoux and Audinat, 2015). In addition, the CD200 protein expressed on the surface of neurons, astrocytes and oligodendrocytes interacts with its receptor CD200R, which is expressed only by macrophages and microglia. The interaction between CD200 and CD200R induces microglia inactivation and maintains microglia in a resting state (Biber et al., 2007). Microglia in AD are initially activated by Aβ formation, through the ability of the pattern recognition receptors (PRRs) to recognize misfolded and aggregated proteins with a consequent trigger of the innate immune response (Tu et al., 2015), leading to migration of the microglia close to the plaques and subsequent phagocytosis of Aβ. When the pro-inflammatory stimulus is chronic, microglia efficacy to bind and phagocyte Aβ decreases and the overall clearance becomes compromised while the immune activation continues (Hickman et al., 2008). The accumulation of Aβ together with chronic release of pro-inflammatory cytokines drives neuronal damage by reducing trophic factors such as brain-derived neurotrophic factor (BDNF) and insulin-like growth factor (IGF; Sheng et al., 1998; Hickman et al., 2008).

The colony stimulating factor 1 (Csf 1) and IL-34 are also important for microglia proliferation during the neurodegenerative process occurring in AD (Gómez-Nicola et al., 2013).

The mechanisms involved in microglia activation induced by Tau are not yet fully elucidated. Recent data show that microglia phagocytose of aggregated Tau, which is targeted to lysosomes, leading to NLRP3 inflammasome activation (Stancu et al., 2019). Moreover, tau interacts with polyglutamine binding protein 1 (PQBP1), which in turn induces the cGAS-STING pathway, leading to microglia activation (Jin et al., 2021).

The microglial myeloid differentiation primary response 88 (MyD88) and the p38 mitogen activated protein kinase (MAPK) signaling pathways are also involved and drive the release of neurotoxins, in a process initiated by the pro-inflammatory cytokine TNF, contributing to the damage of neurons (Michaud et al., 2011; Wang et al., 2015; Schnöder et al., 2016). Moreover, in these conditions, microglia are characterized by a phenotypic change: the retraction of their processes that correlates with an impaired ability to remodel synapses, a phenomenon that contributes to impaired synaptic plasticity observed in AD (Nimmerjahn et al., 2005). All these events contribute to the microgliosis that leads to neurodegeneration (Zhang J. et al., 2021).

Microglia activation leads to the secretion of IL-1β and IL-18, which plays a role in AD progression, as we will describe in more detail in the paragraphs below.

### 3.2. Astrocytes

Astrocytes provide trophic support to neurons and form a protective barrier that isolates neurons from amyloid deposits. Astrocytes are not only essential for the maintenance of neuronal health, but also are an important component implicated in synaptic transmission, they modulate brain energetics and cerebrovascular function (Delekate et al., 2014). In the healthy brain, astrocytes are assembled into dynamic networks. Connexins control this network and their expression is reshaped in AD, leading to a perturbation of the astrocytic network as occurs when astrocytes are activated leading to reactive astrogliosis (Delekate et al., 2014). Besides microglia, astrocytes contribute to the clearance of Aβ but this role is affected in the presence of chronic stress and inflammation. Accumulation of astrocytes, indeed, has been detected in proximity of the Aβ deposits in AD patients (Kuchibhotla et al., 2009). Recently, it has been shown that the functional connectivity of astrocytes is altered early in AD (Shah et al., 2022). Indeed, it is observed a decreased calcium signaling in astrocytes of AD mice before the appearance of Aβ plaques (Shah et al., 2022). Moreover, disruption of astrocyte network in AD affects the cortical neuronal activity, promoting cognitive decline (Lines et al., 2022). The prolonged response of astrocytes to Aβ accumulation promotes neuroinflammation, contributing to nitric oxide (NO) mediated neurotoxicity. Activated astrocytes can participate to the formation of Aβ, as suggested by Rossner et al. in a study on the overexpression of β-secretase (BACE1) in astrocytes affected by chronic stress (Rossner et al., 2005). Moreover, the end-feet of these cells form a lacework of fine lamellae closely linked to the outer surface of the BBB endothelium and basement membrane. Inflammation induces astrocytes proliferation and activation, followed by astrocytes loss and changes in the end-feet structures, affecting the integrity of the BBB (Escartin et al., 2021). Finally, the ability of astrocytes to produce pro-inflammatory prostaglandins and cytokines, such as IL-1β and IL-18, in large quantities during prolonged inflammation negatively alter the BBB protective function, as better reported in the next paragraph (Sofroniew, 2015).

### 3.3. Blood brain barrier

The blood brain barrier (BBB) is one of the main brain barriers that impedes free diffusion between brain and blood and regulates the transport of essential nutrients, ions, and metabolic waste products. It is formed by endothelial cells which are tightly linked by tight junctions (TJs) and it is surrounded by pericytes and astrocytes, which provide support and regulatory functions.

Systemic inflammation has disruptive effects on the BBB integrity, leading to the diffusion of peripheral inflammatory factors into the brain. These factors induce alterations into the brain that in turn may participate to neuronal dysfunction and cognitive decline in AD patients. Among the known factors, the most important are prostanoids and NO. The mediators involved in their release, the Matrix Metalloproteinases (MMPs) and the Reactive Oxygen Species (ROS), activate pathways such as the MAPK, and induce mitochondrial dysfunctions with a subsequent destruction of the BBB integrity (Ralay Ranaivo et al., 2012; Takata et al., 2021). Some miRNAs have a role in the BBB structure and function integrity maintenance. The expression of miR-155 in microvessels is strongly and rapidly upregulated by inflammatory cytokines and alters BBB function by affecting expression of TJs and adhesion components (Lopez-Ramirez et al., 2014). Next to direct morphological changes in the BBB, systemic inflammation can also cause non-disruptive changes that affect BBB functionality (Perrone et al., 2012). Chronic inflammation downregulates the multi-functional efflux transporter permeability glycoprotein (P-gp) and upregulates the expression of the influx carriers responsible for TNF translocation (Roberts, 2008). Systemic inflammation is also responsible for the reduced bulk flow of cerebrospinal and interstitial fluids, resulting in impaired Aβ clearance (Tarasoff-Conway et al., 2015; Mogensen et al., 2021). In AD, the elevated production of TNFα and IL-1β by microglia and astrocytes promotes BBB dysfunction by affecting the TJs, leading to BBB hyperpermeability. In addition, TNFα and IL-1β reduce the expression of the low-density lipoprotein receptor-related 1 (LRP1), which drives the efflux of Aβ from the brain to the blood, resulting in a reduced Aβ clearance (Versele et al., 2022).

## 4. Role of the inflammasome in AD

### 4.1. The NLRP3 complex

We underlined above the crucial role of cytokines and chemokines in the inflammation process. However, the molecular pathways involved are not yet fully characterized. Although it is well documented that IL-1β, IL-6, TNF-α, IL-8, and TGF-β and macrophage inflammatory protein-1a (MIP-1a) are upregulated in AD patients (Domingues et al., 2017), it is not yet fully clarified their role in the onset or progression of AD. Pro-inflammatory cytokines can promote Aβ formation by upregulating BACE1 and APP levels. Further studies are necessary to elucidate the mechanisms induced by pro-inflammatory molecules and their effect in AD progression. Recently, several publications described the role of nucleotide-binding domain, leucine-rich-repeat containing family, pyrin domain-containing 3 (NLRP3) inflammasome in AD (Milner et al., 2021; Barczuk et al., 2022; Sharma et al., 2022). The NLRP3 inflammasome is a multiprotein complex, activated upon infection or stress. The inflammasome is formed by NLRP3 protein and the apoptosis associated speck-like protein containing a caspase recruitment domain (ASC). This macromolecular complex responds to pathogen-associated molecular patterns (PAMPs) and damage-associated molecular patterns (DAMPs; Kelley et al., 2019).

NOD-like receptor family, pyrin domain containing 3 contains an N-terminal pyrin domain (PYD), which clusters upon stimulation, allowing the interaction with ASC and the procaspase-1. Procaspase-1 clustering drives its autocleavage and the formation of the active caspase-1, which induces the cleavage of inactive cytokines pro-IL-1β and pro-IL-18 into their active forms, IL-1β and IL-18, respectively. Notably, enhanced levels of IL-1β induces the production of other cytokines, including IL-6, which in turn promotes the activation of the kinase CDK5 that hyper-phosphorylates tau, further promoting AD progression (Rauf et al., 2022). Furthermore, chronic production of IL-1β enhances brain acetylcholinesterase activity and microglia activation, producing a vitious circle of dysfunction that promotes AD progression by reducing the acetylcholine function and promoting inflammation (Rauf et al., 2022). Chronic IL-1β secretion leads also to astrocytes activation and subsequent production of the pro-inflammatory S100β, further exacerbating neuroinflammation (Rauf et al., 2022). In addition to caspase-1-dependent cytokine production, caspase-1 activates Gasdermin D (GSDMD) by cleaving it, producing a fragment that oligomerizes generating a pore-forming complex that translocates to the plasma membrane leading to release of pro-inflammatory cytokines and pyroptosis, a lytic form of cell death (Yang et al., 2019). Inflammasome activation exacerbates amyloidogenesis, as shown by the study of Venegas et al. (2017), which shows that ASC specks can cross seed Aβ in AD and consequently perpetuate the vicious cycle of brain inflammageing. Intriguingly, NLRP3 inflammasome activation is associated with mitochondrial function, as described in the next paragraph.

### 4.2. Mitochondria alterations in AD

Mitochondria are dynamic double-membrane organelle which undertake several roles from energy production to apoptosis regulation, Ca^2+^ signaling, lipid, and amino acid synthesis. Mitochondrial ATP, produced by aerobic oxidative phosphorylation (OXPHOS), plays an important role for the cell functions. Indeed, enzymatic complexes, present in the inner membrane of the mitochondria, synthesize ATP from ADP and phosphate. However, OXPHOS is a major source of endogenous toxic free radicals, including hydrogen peroxide (H2O2), hydroxyl (HO), and superoxide (O2^−^) radicals that are products of normal cellular respiration (Moreira et al., 2010). Data demonstrate an impairment of the mitochondrial enzymatic complex in AD brain (Castellani et al., 2002). Neurons in AD show striking and significant increase of mitochondrial DNA (mtDNA) localized in the cytoplasm, demonstrating mitochondrial damage (Castellani et al., 2002). ROS overproduction in AD impairs mitochondrial function and reduces the supply of the ATP. Several research demonstrate that oxidative damage occurs before Aβ plaque formation (Nunomura et al., 2001), suggesting that mitochondrial dysfunction plays an early role in AD pathophysiology. Mitochondrial abnormalities appear to be key features during the maturation of AD-like pathology in YAC and C57B6/SJL transgenic mice. In the absence of energy source, abnormal mitochondria produce an excess of free radicals, which can reduce the supply of ATP and contribute to mitochondrial dysfunction in AD (Reed et al., 2008). AD mice show APP mitochondrial localization in cortical neurons, overexpression of oxidative stress markers, deletions in mtDNA, and impaired energy metabolism (Devi et al., 2006). In particular, mitochondrial APP localization inhibits the translocation of COX subunits IV from the cytoplasm to mitochondria, reducing COX activity and increasing H_2_O_2_ levels (Devi et al., 2006). Intra-cerebroventricular injection of Aβ in mice induces H_2_O_2_ production in neocortex mitochondria, leading to neurotoxicity (Morais Cardoso et al., 2002). Aβ also affects the mitochondrial membrane potential (Sbai et al., 2022), affects the mitochondrial fission/fusion balance and mitochondrial distribution (Wang et al., 2020).

Neuronal function is also modulated by the Ca^2+^ concentration and mitochondria are high-capacity Ca^2+^ sinks that control the cytosolic Ca^2+^ loads. Aβ in the presence of Ca^2+^ promotes mitochondria dysfunction by inducing a complete uncoupling of respiration, altering the morphology of mitochondria, decreasing the mitochondrial membrane potential, and affecting the mitochondria capacity to accumulate Ca^2+^ (Moreira et al., 2001). The excessive Ca^2+^ uptake into mitochondria enhances ROS production, which inhibits ATP synthesis and increases the mitochondrial permeability transition pore (PTP). Increased PTP levels promote the release from the mitochondria of various proteins, such as cytochrome *c* and apoptosis-inducing factor (AIF), promoting the initiation of apoptosis by activating the caspase cascade (Hengartner, 2000). Moreover, pro-apoptotic proteins released by mitochondria can translocate into the nucleus, leading to DNA fragmentation, which in turn promote cell death.

### 4.3. Role of mitochondria in activating NLRP3 inflammasome

Activators of NLRP3 induce both NLRP3 deubiquitination and the destabilization of mitochondria membrane potential, its permeabilization, and permeability transition that results in the externalization and release of mitochondria derived molecules (i.e., mitochondrial DNA, ATP, etc.; Liu et al., 2018). These molecules bind NLRP3 and cause its translocation to mitochondria surface, with consequent inflammasome activation, which occurs in the cytoplasm.

A two-step model has been proposed to describe the activation of the NLRP3 inflammasome: a first signal, in response to proinflammatory receptors, induces NF-κB-dependent expression of both proIL-1β and NLRP3 (transcriptional priming) and NLRP3 deubiquitination (non-transcriptional priming); later, a second mechanism triggers the assembly and activation of the inflammasome (Sutterwala et al., 2014). Exception to this general mechanism has been described: the model is not always applicable, with signal I or II missing depending on the molecule activating the NLRP3 inflammasome assembly (Gaidt et al., 2016).

Reactive Oxygen Species have a crucial role in the NLRP3 activation and assembly. Many pathogens and endogenous signals promote ROS generation (Martinon, 2010). Several mitochondrial processes lead to ROS production, which can activate signaling cascades involved in proliferation, apoptosis, and senescence. Antioxidant enzymes such as superoxide dismutase (SOD) and glutathione peroxidase control ROS homeostasis, protecting the cells from the dangerous effects caused by an imbalance in ROS production. When the anti-oxidant systems fail in maintaining the ROS homeostasis, the cells are subjected to oxidative stress and cytotoxicity. Oxidative stress, indeed, activates inflammatory pathways and promotes the formation of pro-mutagenic DNA adducts, creating genetic instability that participate in the progression of various diseases (Sosa et al., 2013; Huang et al., 2016). Considering that NLRP3 and ASC localize within mitochondria upon NLRP3 activators stimulation, studies suggested that NLRP3 activators could induce mitochondrial ROS generation which in turns activate the NLRP3 inflammasome (Zhong et al., 2013; Pang et al., 2021). Studies investigating the effect of ROS scavengers and ROS inhibitors support this hypothesis (Bauernfeind et al., 2011; Alfonso-Loeches et al., 2014; Dai et al., 2017; Liu et al., 2017). Nevertheless, ROS seems to function in the priming step, but not in the activation one (Bauernfeind et al., 2011). Further studies are needed to elucidate the link between ROS and NLRP3 activation (Abais et al., 2015).

### 4.4. Role of NLRP3 in AD

Recent studies demonstrate that NLRP3 inflammasome activation contributes to the pathogenesis of chronic inflammatory or metabolic disorders.

In the AD context, Aβ has been described as priming stimulus to NLRP3 transcription (Nakanishi et al., 2018), however, the molecular mechanisms involved are not yet fully elucidated. Aβ phagocytosis by microglia represents a key step for lysosomal destabilization and consequent release of lysosome content. The assembly of the inflammasome is later promoted by Cathepsin B, a lysosomal proteolytic enzyme, by a still unknown mechanism (Campden and Zhang, 2019). Moreover, Aβ induction of NLRP3 activation leads to GSDMD cleavage and production of a Gasdermin D (GSMD) fragment that oligomerizes at the plasma membrane, resulting in pyroptosis (Han et al., 2020). Another fascinating hypothesis suggests that a negative regulator of NLRP3, NLRP10 (Eisenbarth et al., 2012), reduces NLRP3 assembly. NLRP10 is able to bind ASC, decreasing its availability to interact with NLRP3, reducing the assembly of the inflammasome. In stressful conditions, NLRP10 is degraded by the cathepsin released upon Aβ phagocytosis, leaving ASC free to bind NLRP3 and form a functional inflammasome (Murphy et al., 2014).

Moreover, the release of ATP from dying neurons modulates the activation of NLRP3 inflammasome by activating the P2X7 receptors on microglia, causing K^+^ efflux, ROS generation, and inducing the recruitment of pore-forming pannexin-1. The formation of this channel by pannexin-1 promotes the entry of extracellular molecules, altering the homeostasis of NLRP3 activation (Gombault et al., 2012).

Although several studies investigate the relationship between Aβ and NLRP3, there are only few reports analyzing the link between tau and NLRP3 inflammasome. In 3xTg AD mice model, the inhibition of IL-1β receptor by injection of blocking antibodies results in reduced tau kinase activity and ameliorates the cognitive impairment, implying that NLRP3 inflammasome activation exacerbates tau phosphorylation and subsequent neuronal damage (Kitazawa et al., 2011). Ising and colleagues demonstrate that loss of function of NLRP3 reduces tau hyperphosphorylation and aggregation (Ising et al., 2019). Another study shows that tau activates the NLRP3 inflammasome after being phagocytosed by microglia, further enhancing tau-mediated damage (Stancu et al., 2019). Furthermore, in tau transgenic mice, ASC deletion or NLRP3 inhibition blocks tau-induced pathology (Liang et al., 2022). These data indicate that activation of NLRP3 exacerbate tau pathology through a positive feedback loop, which contributes to AD pathology.

**Figure 1:**
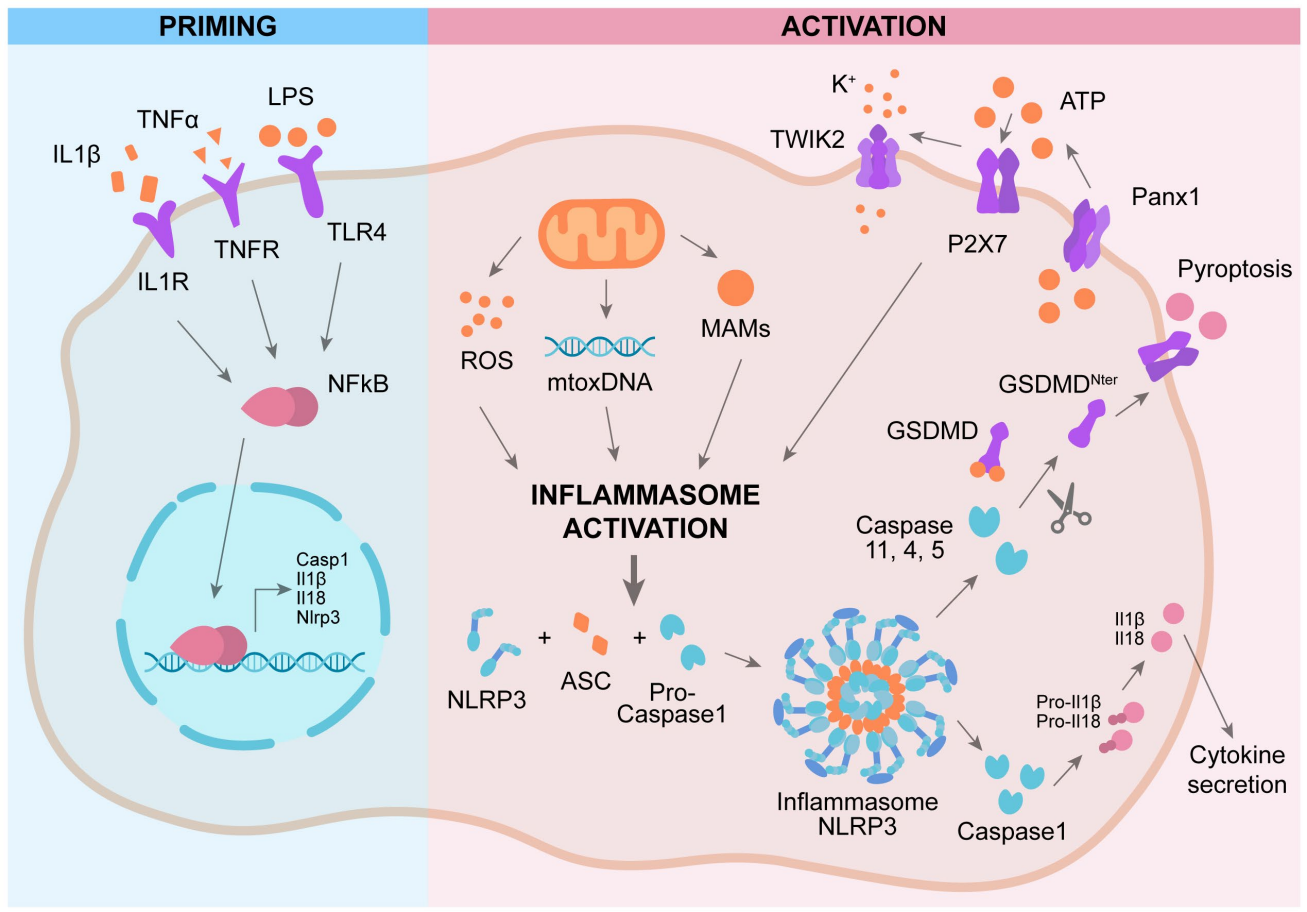
Mechanism of activation of NLRP3 inflammasome. Priming: activation of plasma-membrane receptors induces NFkB activation and subsequent expression of genes involved in NLRP3 cascade. Activation: a second activation of different plasma-membrane receptors promotes the assembly of NLRP3 complex, leading to activation of caspase 1 and subsequent cleavage pf pro-IL1β and GSMD, promoting IL1β secretion and pyroptosis.

While the role of NLRP3 in microglia is well characterized, only recently researchers focused on NLRP3 role in astrocyte. Studies carried out analyzing astrocytic cell lines stimulated with LPS indicate that NLRP3, ASC, and IL-1β are not expressed in astrocytes (Gustin et al., 2015). This observation is supported by data obtained by employing neurosphere-derived astrocytes (Tarassishin et al., 2014). On the other side, Tg2576 transgenic mice, which develops Aβ plaques, show a strong expression of IL-1β in the reactive astrocytes surrounding the Aβ deposits (Apelt and Schliebs, 2001). In agreement, other studies carried out in animal models presenting lesions of the central nervous system demonstrate that astrocytes express NLRP3, ASC, and caspase-1 (Kawana et al., 2013). The mechanism of activation of NLRP3 in astrocytes is still controversial. Recent investigations provide data indicating that GSMD is cleaved by a noncanonical caspase 4 pathway in astrocytes in AD (Moonen et al., 2023). The NLRP3 inflammasome is active also in the BBB, leading to BBB dysfunction (Liu et al., 2022). Knock out of NLRP3 in AD mice (APP/PS1/NLRP3^−/−^ mice) ameliorates memory deficit as well as decreases Aβ production and deposition. These mice show an increment in anti-inflammatory microglia, promoting the phagocytosis and clearance of Aβ (Heneka et al., 2013). NLRP3 inflammasome inhibitor Mcc950 exerts a beneficial effect in a rat model of AD by ameliorating the synaptic plasticity (Qi et al., 2018). Indeed, in this rat model of AD the long-term potentiation (LTP) is impaired and treatment with Mcc950 ameliorates the LTP impairment (Qi et al., 2018). Enhanced NLRP3 activation promotes a chronic inflammatory state leading to tau hyperphosphorylation, NFT formation, and synaptic dysfunction (Heneka, 2017). Knock out of NLRP3 reduces tau hyperphosphorylation and ameliorates spatial memory impairment in Tau22 mice (Ising et al., 2019). In early phase AD patients, the levels of IL-1β and caspase 1 activity are enhanced, confirming the over activation of NLRP3 as early event in AD (Venegas et al., 2017). Peripheral blood mononuclear cells (PBMCs) derived from AD and amnestic mild cognitive impairment (aMCI) patients shows elevated levels of NLRP3, IL-1β and caspase 1, showing the presence of systemic NLRP3 activation and inflammation in aMCI and AD patients (Rui et al., 2021; Figure 1).

### 4.5. Other inflammasomes and their role in AD

There exist 22 human NLR proteins which can bind various caspases involved in the production of inflammatory cytokines, more frequently caspase-1. NLRs can bind the caspases either directly or via the ASC adaptor, an apoptosis-associated speck-like protein containing CARD and PYD domains. The NLR type of inflammasomes can be subdivided on the basis of their structures and assembly model into the NLRA, NLRB, NLRC (containing, e.g., NOD1, NLRC4, NLRC5, and NLRX), and NLRP (e.g., NLRP1 and NLRP3) subfamilies. The NLRP3 inflammasome is considered a predominant element in the inflammatory process, but the activation of other inflammasomes is also induced by mitochondria dysfunction. Indeed, mitochondrial DNA released in the cytoplasm activates the NLRC4 (Jabir et al., 2015), the NLRP3 and the AIM2 inflammasomes (Korhonen et al., 2021).

Recent findings indicate that NLR family CARD Domain Containing 4 (NLRC4), AIM2 (Interferon-inducible protein AIM2), and NLR Family Pyrin Domain Containing 1 (NLRP1) are also linked to mitochondrial dysfunction and appear to play a key role in neuronal diseases (Patergnani et al., 2021). In a rat model of AD, obtained by intracerebral injection of streptozotocin (STZ), NLRC4 expression is increased as well as IL-1β production, while the expression of NLRP1, NLRP3, and AIM2 is unvaried compared to control rats, suggesting that NLRC4 promotes inflammation in this AD rat model (Saadi et al., 2020). NLRP1 levels are significantly increased in the brain of AD patients and NLRP1 genetic variants are associated with increased risk of AD (Pontillo et al., 2012). NLRP1 is expressed in neurons, where it activates caspase 1, which in turn activates caspase 6, leading to IL-1b production (Kaushal et al., 2015). In the APP/PS1 AD mice model, NLRP1 is upregulated and NLRP1 silencing in this AD model reduces neuronal pyroptosis and cognitive impairment (Tan et al., 2014). An *in vitro* model of AD is obtained by treating astrocytes with palmitate, which activates a pro-inflammatory response and IL-1β production, which in turn leads to enhanced Aβ production in neurons (Liu and Chan, 2014). In this AD model, palmitate induces the activation of ice protease-activating factor (IPAF)-which interact with ASC, leading to IL-1β production. Silencing of IPAF in astrocytes decreases IL-1β secretion and diminishes Aβ production in neurons (Liu and Chan, 2014).

## 5. Role of Drp1 in AD inflammation

### 5.1. Structure and function of Drp1

The Dynamin-related protein 1 (Drp1), is a dynamin-*like* GTPase protein required to modulate the dynamics of fusion and fission of mitochondria, in particular it is required for the fission of mitochondria (Kandimalla and Reddy, 2016). Drp1 shows various subcellular localizations: cytoplasm, mitochondria, Golgi and peroxisomes. It is encoded by the Dnm1 gene, which is located on 12p11.21 chromosome in mice and on 11q23 in human (Oliver and Reddy, 2019). The RNA transcribed from the Dnm1 gene can be subjected to alternative splicing, which give rise to various isoforms of Drp1 (Oliver and Reddy, 2019). The largest isoform (variant 1) is constituted by 736 amino acids and its calculated molecular mass is 81.6 kDa. Exon 15 is spliced out in variant 2 isoform, producing a protein with 710 amino acids; both exons 15 and 16 are spliced out in variant 3, resulting in a protein with 699 amino acids; variant 4 has 725 amino acids; variant 5 is constituted by 710 amino acids and variant 6-which is present only in neurons-is formed by 749 amino acids (Reddy et al., 2011).

Dynamin Related Protein 1 is characterized by the presence of highly conserved domains. It contains four GTPase domains: the N-terminal GTPase domain, the middle domain, a variable or B domain, and the C-terminal GTPase effector domain. Drp1 has two major phosphorylation sites: CDK phosphorylates in S579, a PKA site is located at S600 in Drp1 isoform 3 (Kandimalla and Reddy, 2016; Oliver and Reddy, 2019).

The mitochondrial fusion and fission processes are controlled during cellular activation by several proteins and not yet fully elucidated mechanisms (Sharma et al., 2019). Current research has demonstrated that Drp1, Fis1, Miro, Opa1, Mfn1, Mfn2, Mid49, Mid51, and Mff are associated with mitochondrial dynamics, morphology, distribution, and function (Kandimalla and Reddy, 2016; Oliver and Reddy, 2019). Drp1 is a key regulator of mitochondrial fission. Drp1 activation induces its translocation from cytoplasm to the mitochondria outer membrane, where it interacts with the fission protein 1 (Fis1), promoting a contraction and a split of the mitochondria. Ramonett et al. (2022), based on proteomic interactome analysis, reported that Drp1 interacts with GAIP/RGS19-interacting protein (GIPC) through its atypical C-terminal PDZ-binding motif. Next, GIPC mediates the actin-based retrograde transport of Drp1 toward the perinuclear mitochondria to enhance their fission. GIPC (Ramonett et al., 2022).

Notably, the balance between mitochondrial fusion and fission is crucial to maintaining a physiological dynamic regulation of mitochondrial function. Downregulation of Drp1 promotes fusion. Loss of Drp1 triggers genome instability, cell cycle arrest and initiates the DNA damage response by disrupting the mitochondrial dynamics and distribution (Qian et al., 2012). Drp1 is enriched at neuronal terminals and involved in synapse formation and synaptic sprouting. Drp1 can be phosphorylated at different sites, which play opposite functions, leading to either increased fragmentation or enhanced fusion of mitochondria (Oliver and Reddy, 2019).

Dynamin Related Protein 1 is involved in the mechanisms underlying various diseases, such as myocardial ischemia/reperfusion injury, heart failure, and cancer. Indeed, tumor progression can be affected by Drp1 mediated regulation of mitochondrial metabolism. In agreement, *in vitro* studies indicate that phosphorylation of Drp1 promoting mitochondrial fission inhibits mitochondrial oxidative phosphorylation and enhances aerobic glycolysis, which promotes growth and metastasis in cancer cells. Drp1 silencing causes mitochondrial elongation and significantly suppresses the metastatic abilities of breast cancer cells (Rodrigues and Ferraz, 2020).

Moreover, Drp1 modulates the production of the cytokine IL-1β, IL-6, and IFN-β in response to immune stimulation and infection (Tiku et al., 2020). Indeed, swine influenza virus (SIV) infection leads to Drp1 phosphorylation at serine 579 and subsequent mitochondrial fission and IL-1β production in alveolar macrophages (Park et al., 2018). In particular, SIV infection induces ROS formation as well as the activation of the receptor-interacting protein kinase 1 (RIPK1), which in turn phosphorylates Drp1. RIPK1-dependent Drp1 phosphorylation is necessary for mitochondrial fission and ROS release, which in turn activate the NLRP3 inflammasome and subsequent IL-1β production (Park et al., 2018).

**Figure 2:**
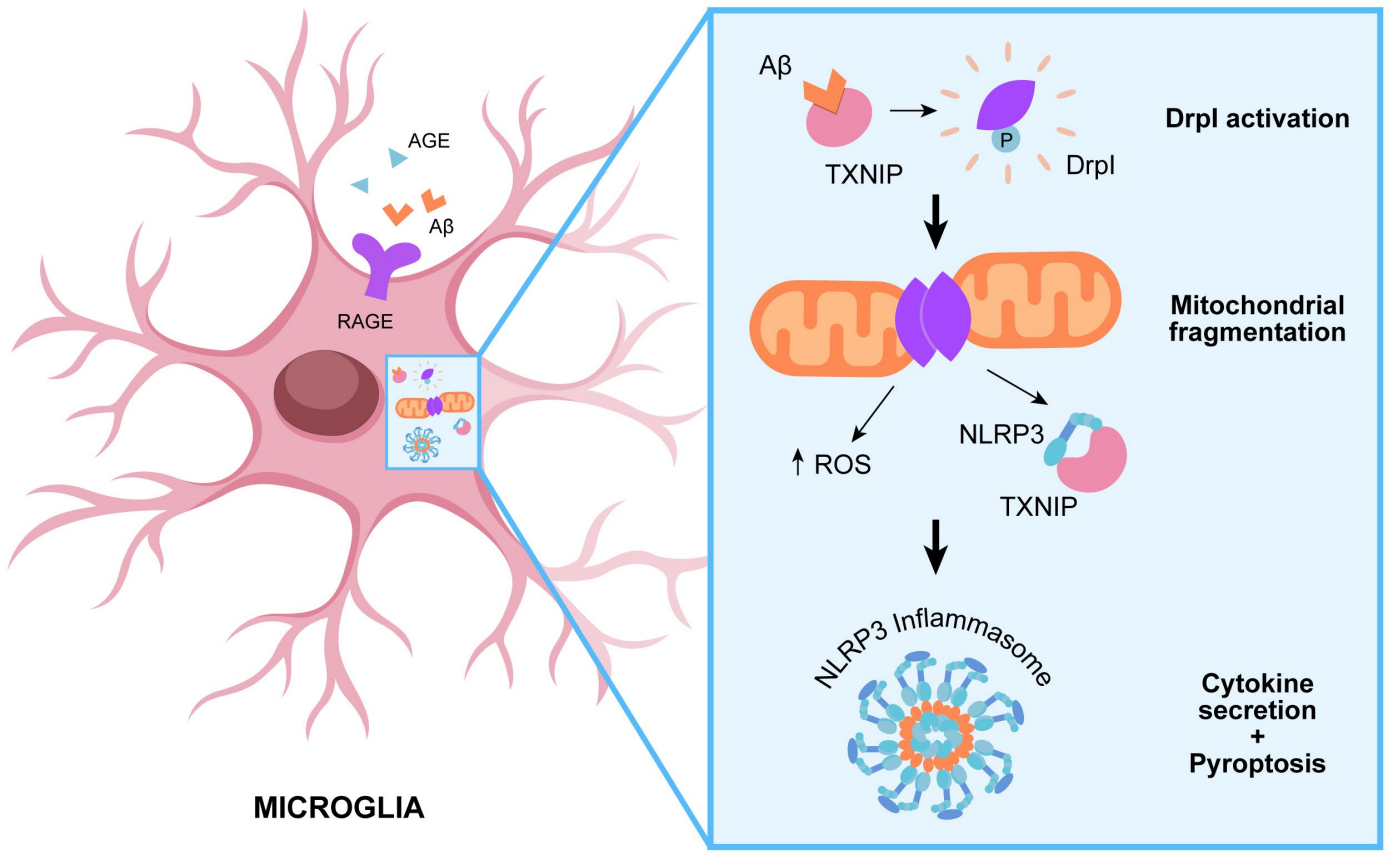
Role of Drp1 in promoting NLRP3 assembly. TXNIP-mediated recruitment of DRp1 to mitochondrial results in aberrant mitochondrial fission and ROS production, which in turn promote NLRP3 assembly and subsequent inflammatory cascade.

In agreement, it is emerging a role of Drp1 in modulating NLRP3 activation (Park et al., 2015). Parkin et al. provide a molecular insight into the relevance of mitochondrial dynamics in potentiating NLRP3 inflammasome activation, leading to aberrant inflammation. Knockdown of dynamin-related protein 1 (Drp1) induces aberrant mitochondrial elongation, promoting a marked increase in NLRP3-dependent caspase-1 activation and IL-1β secretion in mouse bone marrow-derived macrophages (Park et al., 2015). Several studies highlight the importance of Drp1 for mitochondrial balance between fission and fusion, which is modulated in response to infection to enhance macrophage effector function. Indeed, infection with bacteria commonly results in mitochondrial fragmentation, whereas viral infection often leads to mitochondrial fusion in macrophages (Tiku et al., 2020). Surprisingly, both enhanced fission and fusion can promote NLRP3 activation through different pathways. It seems that mitochondrial fission is implicated in IL-1β production following bacterial infection. Indeed, mitochondrial fission results in an increased cytosolic level of mitochondrial DAMPs, such as mitoDNA or mitoROS, inducing NLRP3 inflammasome activation (Park et al., 2015). On the other hands, the cellular response to cytosolic viral RNA promotes mitochondrial fusion and the activation of the mitochondrial antiviral-signaling (MAVS) protein, which in turn activates the NLRP3 inflammasome (Park et al., 2013; Figure 2).

### 5.2. Role of Drp1 in AD

Alzheimer Disease is characterized by a diminished expression of genes involved in mitochondrial biogenesis, affecting the mitochondrial homeostasis and producing defective mitochondria biogenesis (Kandimalla et al., 2018; Manczak et al., 2018). Recent data show that Drp1 expression is significantly enhanced in the brain of AD patients and AD mice compared to healthy human controls and wild type mice, as well as Drp1 expression increases in neurons exposed to Aβ peptides *in vitro* (Medala et al., 2021; Bera et al., 2022). Moreover, a study carried out in an *in vitro* AD model revealed an enhanced expression of genes involved in mitochondrial fission (such as Drp1 and Fis1) and a decreased expression of those related to mitochondrial fusion (such as Mfn1 and Mfn2), altering the mitochondria dynamics and affecting the synaptic function (see below). These observations are corroborated by the analysis of post-mortem brains of AD patients, where the expression levels of Opa-1, Mfn1, and Mfn2 are decreased, while Fis1 is significantly increased. Notably, Aβ induces the S-nitrosylation of Drp1, leading to its hyperactivation and subsequent excessive mitochondrial fragmentation, which in turn generates ROS, leading to synaptic damage (Nakamura et al., 2010). Notably, AD is characterized by enhanced mitochondrial fission, suggesting that Drp1 plays a role in AD (Baek et al., 2017). In agreement, treatment of hemizygous APP/PS1 mice with mitochondrial division inhibitor 1 (Mdivi-1), an indirect inhibitor of Drp1, ameliorates anterograde mitochondrial transport, oxidative stress, and synaptic damage (Baek et al., 2017). Mdivi-1 ameliorates mitochondria fragmentation, distribution, and function also in CRND8 mice (APP strain; Wang et al., 2017). Double transgenic mice obtained crossing heterozygote Drp1 (+/−) mice with AD mice (Tg2576 strain) reveals that partial reduction of Drp1 is beneficial by ameliorating mitochondrial dysfunction, reducing Aβ production, increasing mitochondrial biogenesis and enhancing synaptic activity (Manczak et al., 2016). Reduction of Drp1 is protective also against mutant Tau-induced neuronal dysfunction in AD. A double transgenic mice obtained by crossing Drp1 (+/−) mice with Tau mice (P301L strain) show reduced mitochondrial dysfunction, decreased Tau phosphorylation, enhanced mitochondrial biogenesis and synaptic activity (Kandimalla et al., 2016). Drp1 interacts directly with Aβ in neurons of AD patients, in AD transgenic mice, and also *in vitro* in neurons derived from AD transgenic mice, leading to mitochondrial dysfunction and synaptic damage (Manczak et al., 2011). Tau plays a critical role in the assembly, stabilization, and modulation of microtubules, which are important for the normal function of neurons and the brain. Drp1 interacts directly also with phosphorylated Tau, further inducing neuronal dysfunction (Manczak and Reddy, 2012).

Many researchers have demonstrated that mitochondrial dysfunction can promote energy impairment in AD. Thus, Drp1-induced excessive mitochondrial fragmentation and defective transport of mitochondria to synapses, leads also to reduced synaptic ATP production and subsequent synaptic dysfunction (Pradeepkiran and Reddy, 2020). Aβ accumulation affects mitochondrial dynamics also in astrocytes, inducing a shift in the metabolic pathways (Zyśk et al., 2023). In human induced pluripotent cell (hiPSC)-derived astrocytes treated with Aβ it is observed an increased Drp1 phosphorylation, which localizes in lipid droplets and is secreted in extracellular vesicles (EV) and transported in the surrounding astrocytes through tunneling nanotubes (TNTs), enhancing the mitochondrial OXPHOX and increasing the glycolysis, switching toward fatty acid β oxidation for energy production (Zyśk et al., 2023). Astrocytic EVs (called astrosomes) derived from AD mice (5xFAD) contain Aβ (Elsherbini et al., 2020). 5xFAD-derived astrosomes are transported to mitochondria when added to neuronal cells *in vitro*, leading to enhanced Drp1 expression, altering the mitochondria dynamics and promoting caspase 3 activation, which in turn induces neuronal cell death (Elsherbini et al., 2020).

Oligodendrocytes are required for the myelination process, which is strongly dependent on glycolysis, which requires an efficient mitochondrial function. Thus, hyperactivated Drp1 can affect myelination by disrupting the mitochondrial activity. Notably, mature oligodendrocytes both in human AD patients and AD mice show inflammatory injuries associated with the NLRP3 inflammasome (Zhang et al., 2020). NLRP3 can be activated by components of glycolytic pathway and ROS, suggesting the role of Drp1 also in promoting the inflammatory process in AD. In agreement, excessive activation of Drp1 causes a glycolytic dysfunction in AD mice, which in turn activates NLRP3 inflammasome and subsequent pyroptosis. Knock down of Dp1 in oligodendrocytes abolishes NLRP3-induced inflammation and ameliorates myelin loss (Zhang et al., 2020). These data demonstrate that Drp1-promoted mitochondrial dysfunction leads to inflammation, which plays a key role in myelin loss and neuronal dysfunction.

Reactive Oxygen Species is the major factor responsible of imbalance in mitochondria function. Several recent findings report that advanced glycation end products (AGEs) participate in inflammation and neurodegenerative diseases (Perrone et al., 2012). AGE and its receptor RAGE are over expressed in AD brain samples, induce ROS production and mitochondria dysfunction. AGEs can influence mitochondrial dynamics by impairing fusion-fission balance, with a significant increment of mitochondrial fission in AD (Pradeepkiran and Reddy, 2020). AGEs contribute significantly to mitochondria dysfunction, by upregulating the expression of fission proteins Drp1 and Fis1 and down-regulating fusion proteins Mfn1, Mfn2, and Opa1. AGEs are also involved in abnormal APP processing and Aβ production. Indeed, AGEs activate RAGE, which in turn enhances the expression of BACE 1, a key enzyme in APP processing that initiates Aβ production (Perrone et al., 2012). Both AGE and Aβ trigger RAGE activation, which induces redox sensitive pathways by activating the transcription factor nuclear factor kappa B (NFkB), which promotes the expression of pro-inflammatory genes (Perrone et al., 2012). These pathological pathways promote an inflammatory cascade, which lower glucose consumption, decreases ATP levels, and downregulates mitochondrial activity, ultimately promoting neuronal death (Sivitz and Yorek, 2010). Oxidative stress caused by RAGE triggering promotes neuroinflammation and enhances Aβ levels into the brain by enhancing the rate of Aβ influx, leading to a vicious circle of RAGE activation following interaction with Aβ and further promoting neurodegeneration (Perrone et al., 2012). RAGE promotes inflammation also by inducing the expression of Thioredoxin-Interacting Protein (TXNIP), which is an β-arrestin-containing protein that can bind to and inhibit the antioxidant protein thioredoxin (TXN), promoting oxidative stress (Perrone et al., 2009; Sbai et al., 2010). TXNIP is over expressed in the brain of AD patients and in several AD mice models and recent data suggest that it participates in AD pathophysiology (Melone et al., 2018; Nasoohi et al., 2018a,b; Wang et al., 2019; Tsubaki et al., 2020; Perrone and Valente, 2021; Zhang M. et al., 2021). TXNIP plays a key role in modulating the cell and body glucose homeostasis (Perrone and Valente, 2021). Notably, TXNIP plays a key role in activating the NLRP3 inflammasome (Schroder et al., 2010) and its association with NLRP3 has been shown also in AD (Li et al., 2019; Sbai et al., 2022). Sbai et al. demonstrate that TXNIP drives the transport of Aβ to mitochondria in microglia both *in vivo* AD mice (5xFAD strain) and *in vitro* primary microglia, leading to Drp1 translocation to mitochondria, leading to mitochondria dysfunction, ROS production and NLRP3 activation and subsequent production of cytokines and pyroptosis of microglia, showing that it is an early even in AD, occurring in the early phases of AD when the first signs of cognitive alterations occur (Sbai et al., 2022). Drp1 appears to being transported to mitochondria in a complex together with TXNIP and Aβ, resulting in Drp1 activation. Silencing of TXNIP or blockade of RAGE reduces Aβ transport from the cellular surface to mitochondria in microglia, restores mitochondrial functionality, mitigates Aβ toxicity, inhibits NLRP3-induced inflammation and blocks pyroptosis *in vivo* AD mice and *in vitro* primary microglia (Sbai et al., 2022).

Up until now it has been demonstrated that there is a link between enhanced Drp1 activation and NLRP3 activation. We cannot exclude that Drp1 may modulate the assembly/function of other inflammasome complexes other than NLRP3. However, the data published so far in AD and other pathologies, such as cancer, report a link only between Drp1 and NLRP3 inflammasome. Further studies are needed in order to unveil the role of Drp1 in altering the mitochondrial function and activating inflammasome complexes other than NLRP3. However, the studies described above clearly show the relevance of Drp1 in linking the mitochondrial function to the induction of inflammation.

## 6. Conclusion

Several data are indicating a key role of Drp1 in promoting neurodegeneration by altering mitochondrial function in neurons. However, recent data are underline the role of Drp1 in promoting the activation of NRP3 in not neuronal cells, leading to inflammatory process that in turn leads to neuronal dysfunction. These data support the relevance of Drp1 in promoting mitochondrial dysfunction as an early even in AD. In addition, Drp1 links mitochondrial dysfunction to NLRP3 activation and the subsequent inflammatory cascade. In addition, these data demonstrate the pathological effect of inflammation as early even in AD. Indeed, inflammation occurs before the detection of neuronal dysfunction in all AD mice models. We described publications demonstrating that Drp1 promotes early inflammatory alterations in oligodendrocytes and microglia, further promoting neurodegeneration. These data are essential for the development of innovative strategies aimed to prevent and cure AD at the early stages.

## Author contributions

OS, VB, ST, and LP wrote the manuscript. OS, VB, ST, JM, and CV corrected the manuscript. All authors contributed to the article and approved the submitted version.

## Funding

This work was funded by the European Union’s Horizon 2020 research and innovation program under the Marie Skłodowska-Curie grant agreement No. 956070 and by the European Union’s Horzion Europe research and innovation program under the Marie Skłodowska-Curie grant agreement No. 101072515 to CV.

## Conflict of interest

OS is founder and CEO of Caminnov sas. LP plays a consulting role for Caminnov sas.

The remaining authors declare that the research was conducted in the absence of any commercial or financial relationships that could be construed as a potential conflict of interest.

## Publisher’s note

All claims expressed in this article are solely those of the authors and do not necessarily represent those of their affiliated organizations, or those of the publisher, the editors and the reviewers. Any product that may be evaluated in this article, or claim that may be made by its manufacturer, is not guaranteed or endorsed by the publisher.
